# Sacrococcygeal Yolk Sac Tumor in a Two-Year-Old Girl With Multiple Metastases: A Case Report

**DOI:** 10.7759/cureus.29056

**Published:** 2022-09-11

**Authors:** Didik S Heriyanto, Vincent Lau, Vincent Laiman, Bambang Ardianto

**Affiliations:** 1 Anatomical Pathology, Faculty of Medicine, Public Health, and Nursing, Universitas Gadjah Mada, Sleman, IDN; 2 Child Health, Faculty of Medicine, Public Health, and Nursing, Universitas Gadjah Mada, Sleman, IDN

**Keywords:** yolk sac tumor, sacrococcygeal, multiple metastases, malignant germ cell tumor, immunocytochemistry, alpha-fetoprotein

## Abstract

Sacrococcygeal yolk sac tumor (YST) is an infrequent extra-gonadal malignant germ cell tumor (GCT) that occurs exclusively within the first two years of life. A two-year-old girl came with a massive mass on her left buttock, which continued to grow, and within three months had become extremely large and hindered her from walking. Physical examination revealed a sacrococcygeal mass of 15 cm in diameter. Multislice CT showed an intraluminal inferior cava vein mass extending into the pelvic cavity with coccygeal osseous destruction, pulmonary metastasis, and multiple hepatic metastases. Laboratory data revealed elevated tumor marker values for alpha-feto-protein (AFP), lactate dehydrogenase (LDH), and Ca-125. Cytopathology following fine needle aspiration biopsy evaluation of the smear sample revealed a cellular tumor with pseudo glandular, microcystic, and solid patterns. The cytopathology did not show pathognomic findings. An immunocytochemistry (IHC) examination of the cell block showed a positive result for anti-AFP antibody. The patient was diagnosed and treated with chemotherapy for a sacrococcygeal YST. Clinical follow-up on the fourth month showed that the tumor had shrunk to 4 cm in size. Laboratory follow-up data after four months showed significant improvement. Unfortunately, the patient passed away on the seventh cycle of chemotherapy due to lung and hepatic metastases.

## Introduction

Malignant germ cell tumors (GCTs) account for 3%-4% of all pediatric neoplasms. This malignancy primarily affects young individuals' testes and ovaries. Less than one case per million people per year are malignant GCTs. Malignant GCTs may develop in gonadal and extra-gonadal sites. Malignant ovarian GCT affect women and accounts for 3%-5% of all ovarian cancers. Only 2%-5% of malignant GCTs can originate from extra-gonadal sites and spread axially. Outside of the ovaries, the sacrococcygeal area is one of the most common places for tumors to form [1].

In children, sacrococcygeal teratoma (SCT) accounts for 40% of all GCT cases and 78% of all extragonadal and extracranial GCTs [2-3]. SCT is the most commonly diagnosed fetal neoplasm, with an incidence of one in every 27,000 pregnancies. Females are more likely than males to have SCT, with a ratio of 3-4:1.5 The malignant elements of SCT increase with the age of conception. This malignant degeneration results in the formation of a YST, which is rarely found on its own. Sacrococcygeal YST occurs exclusively within the first two years of life [4-5].

## Case presentation

A two-year-old girl came with a massive mass on her left buttock. The patient had a history of falling at home, resulting in injury. The family brought her to an alternative medicine practitioner for treatment with no significant improvement. The family noticed a small lump on her left buttock, which continued to grow, and within three months had become extremely large and hindered her from walking. Due to the growing mass, her family took her to the hospital. The patient had no family history of malignancy. The doctor suspected malignancy and decided to refer the patient to a national referral hospital to get comprehensive management.

**Figure 1:**
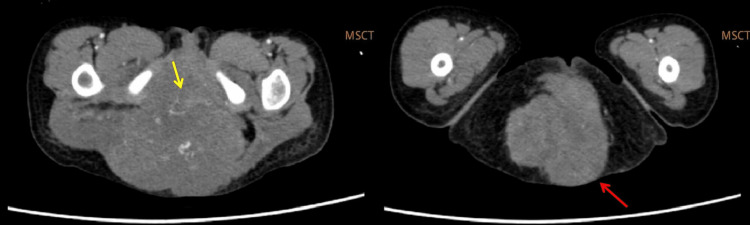
MSCT showed an intraluminal inferior cava vein mass expanding into the pelvic cavity (yellow arrow) and gluteal region with coccygeal osseous destruction (red arrow) with early suspicion of leiomyosarcoma. MSCT, multislice computed tomography

**Figure 2:**
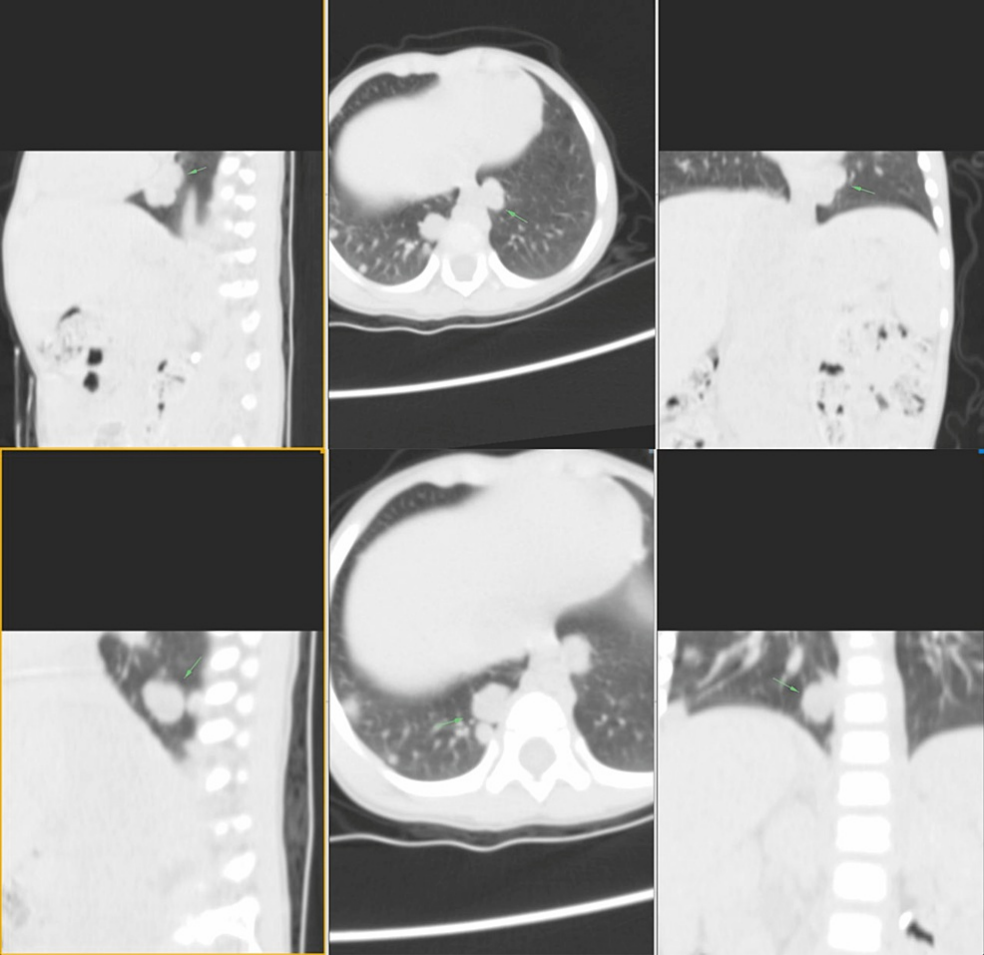
MSCT showed bilateral nodular-type pulmonary metastases. The green arrow showed hyperdense lesion in bilateral pulmonary inferior lobe visualized as nodule with firm borders, regular edges, diameter of 1.1 cm, with increased post-contrast density. MSCT, multislice computed tomography

**Figure 3:**
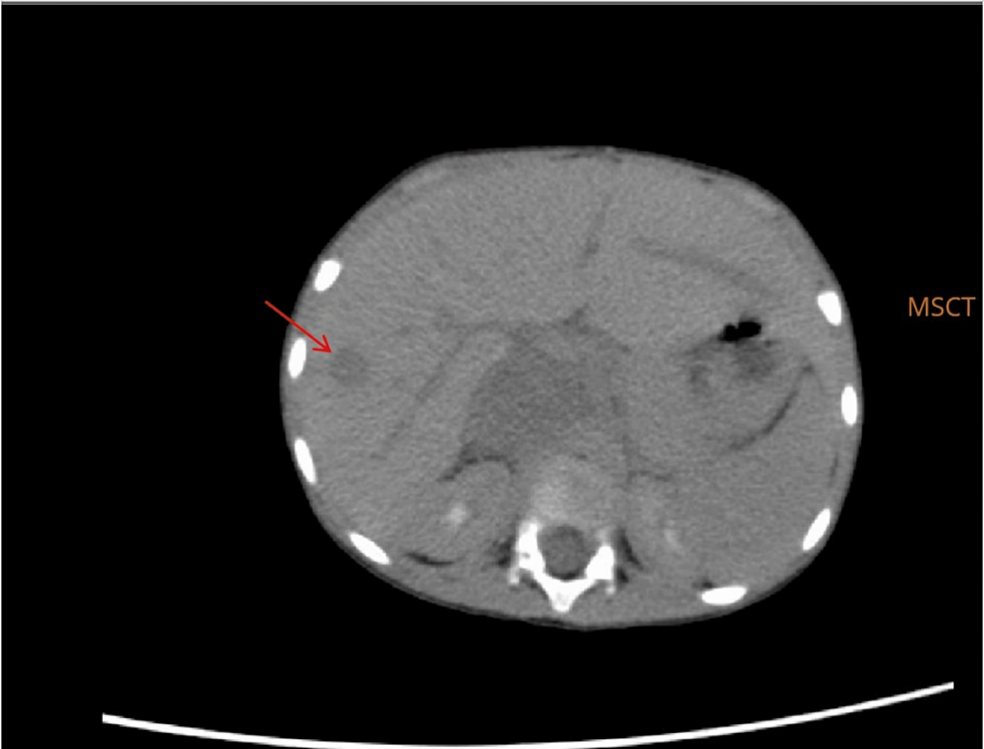
MSCT showed hepatic metastasis. The red arrow showed a hypodense lesion in the segment VI of the liver that is round in shape with irregular borders. MSCT, multislice computed tomography

A physical examination revealed a sacrococcygeal mass of 15 cm in diameter, which is still growing. Multislice CT (MSCT) showed an intraluminal inferior cava vein mass expanding into the pelvic cavity and gluteal region with coccygeal osseous destruction and suspicion of leiomyosarcoma (Figure 1). Multiple metastases were found, consisting of bilateral nodular-type pulmonary metastases (Figure 2) and hepatic metastasis (Figure 3). Laboratory data revealed elevated tumor marker values for alpha-feto-protein (AFP) at 883 U/L (normal reference values: 0-8.3 IU/mL); elevated level of lactate dehydrogenase (LDH) at >50000 IU/mL (normal reference values: 105-333 U/L), and elevated level of Ca-125 at 47.15 U/mL (normal reference value: 0-35 U/mL). Beta-HCG (human chorionic gonadotropin) was normal at 26.1 mIU/mL (normal reference value: <1400 mIU/L).

**Figure 4:**
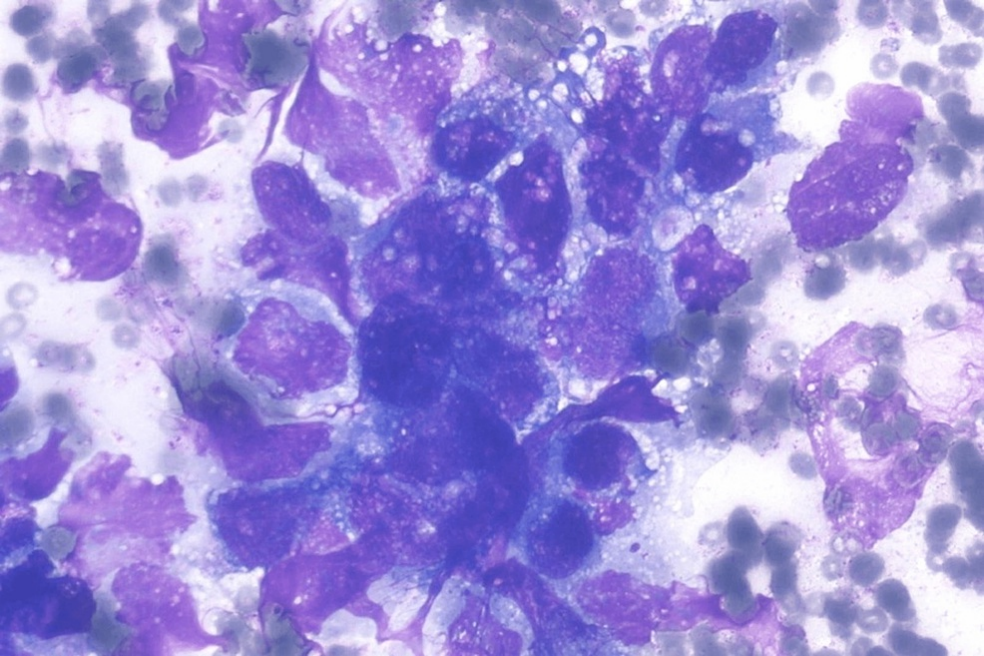
Photomicrograph of a yolk sac tumor composed of large bizzare cell, vacuolated cytoplasm, with myxoid background. (Diff qwikk, 400x).

**Figure 5:**
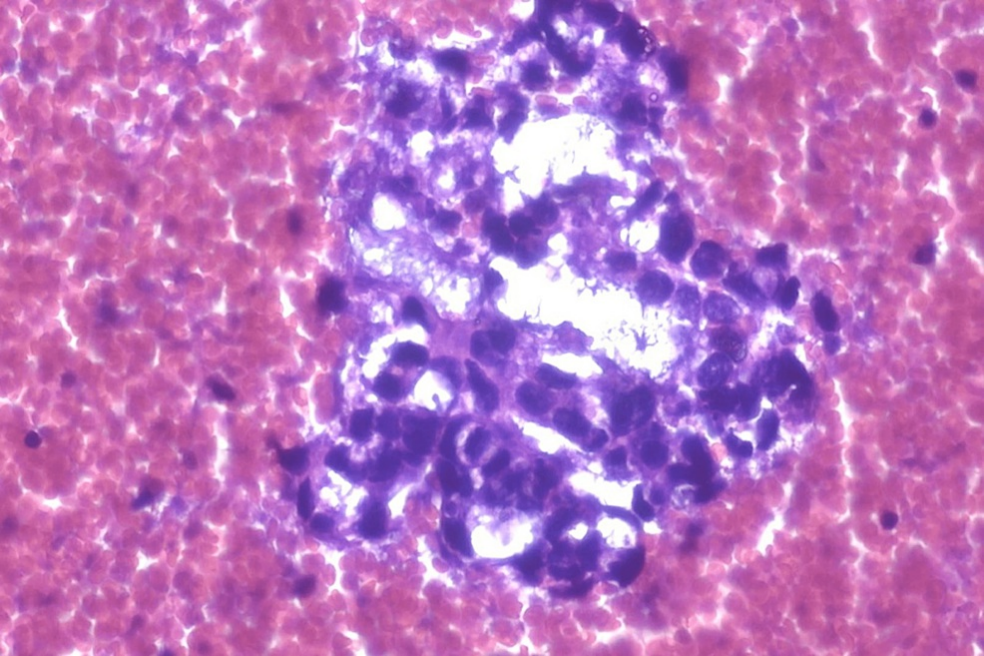
A microscopic exam of the cell block sample revealed a reticular pattern with myxoid masses (Cell block, HE, 400x). HE, hematoxylin-eosin

**Figure 6:**
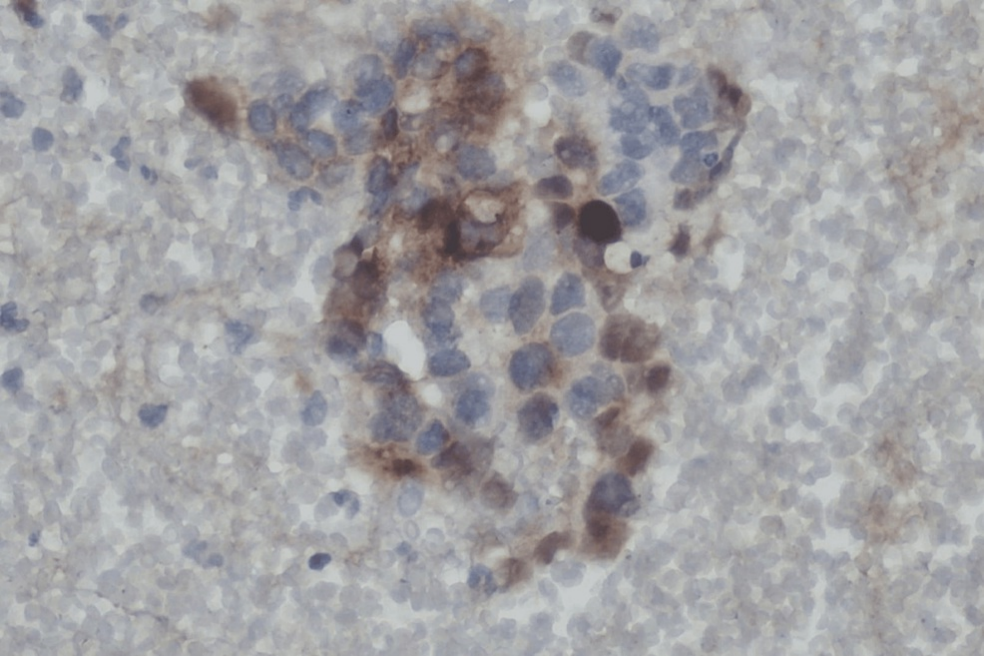
Immunocytochemistry examination of the cell block showed a positive result for anti-AFP antibody (cell block, ICC anti-AFP antibody, 400x). AFP, alpha-feto-protein; ICC, immunocytochemistry

An ultrasound-guided fine needle aspiration biopsy (FNAB) was conducted, followed by a microscopic examination. Cytopathology evaluation of the smear sample revealed a cellular tumor with pseudo glandular, microcystic, and solid patterns. The background of the smear sample revealed diffusely scattered erythrocytes with myxoid areas. Polymorphic cells with vacuolated cytoplasm, large nuclei, and some bizarre, irregular membranes with prominent nucleoli were also found (Figure 4). A microscopic exam of the cell block sample revealed a reticular pattern with myxoid masses (Figure 5). The microscopic finding on the FNAB sample is consistent with the cytopathological finding of the sacrococcygeal YST. Immunocytochemistry (IHC) examination of the cell block showed a positive result for anti-AFP antibody (Figure 6).

The patient was diagnosed and treated for a sacrococcygeal YST. The oncology consultant selected a chemotherapy regimen consisting of cisplatin, etoposide, and bleomycin. The patient came in four months later after going through four cycles of chemotherapy. The tumor had shrunk to 4 cm in size, with a remnant of distant metastases in both the lung and the liver. Laboratory follow-up data on the fourth cycle (fourth month) revealed: elevated AFP level at 21.6 UI/mL (normal reference value: 0-8.3 IU/mL); normal LDH level at 257 U/L(normal reference value: 105-333 U/L), and normal beta-HCG level at 0.2 mIU/mL (normal reference value: <1400 mIU/L) respectively. The laboratory data showed improvement due to the chemotherapy. Unfortunately, the patient passed away on the seventh cycle (seventh month) of chemotherapy. The patient most likely passed away due to lung and hepatic metastases.

## Discussion

In children, a sacrococcygeal mass can lead to multiple diagnoses. It can be a congenital lesion such as meningomyelocele or other neural tube defects, an inflammatory lesion such as seen in rectal abscess, a benign or malignant GCT, or even rhabdomyosarcoma [6]. Malignant GCT affects the gonads (ovary or testis) and extragonadal sites (sacrococcygeal, mediastinum, pineal, and retroperitoneum) [7]. Only 2%-5% of GCT cases are extra-gonadal. SCT is most commonly found in the sacrococcygeal region [1]. Yolk sac tumor (YST) is another GCT that is often found together with SCT. YST is always malignant, and it is extremely rare for it to occur alone [8]. In 2015, Ben Nsir reviewed and reported that sacrococcygeal YST only occurs in children two years of age and younger [9].

Any mass in the sacrococcygeal region could cause a massive mass on the left buttock as clinical presentation. Constipation or swelling of the buttocks are the most common YST complaints, according to the literature. It is also always malignant, which means that the time between the onset of symptoms and hospital admission is often short, and the lesion has most likely spread [8].

Merchant and Stewart published a case report in 2010 about a sacrococcygeal YST in an infant who presented with fluid collection that was initially treated as an abscess. Sacrococcygeal YST appeared as a protruding, growing sacral mass in the body's midline. It is important to note that YST may have different clinical manifestations during the initial assessment and a clinician should not rule out YST as an initial diagnosis [10].

The MSCT showed that the mass is expanding into the pelvic cavity and is destructive to the bone. We can infer that the mass is malignant, suggesting a malignant GCT or rhabdomyosarcoma or leiomyosarcoma. It also showed nodular-type pulmonary and multiple hepatic metastases, supporting the tumor's malignancy. Imaging as a diagnostic tool for YST is surprisingly difficult. In this particular case, it was initially suspected as leiomyosarcoma. SCTs can be diagnosed by solid and cystic tumors with necrotic and hemorrhagic areas. Lung metastasis is common. It is unique that the patient has multiple hepatic metastases. Hepatic metastasis has only been reported in a case report of an abdominal YST by Chen et al. in 2019. It spreads hematogenously in over half of pediatric patients [11].

Laboratory data revealed very high levels of alpha-feto-protein (AFP); a very high level of LDH and high level of Ca125; while beta-human chorionic gonadotropin (β-HCG) levels were within normal limits. High serum AFP may indicate hepatic disorders and malignancies in children, including hepatocellular carcinomas, GCTs, liver metastases, and pancreatoblastoma [12]. Both YST and mixed GCT can have high AFP level. Higher blood LDH levels may indicate colorectal, lung, or GCTs. In cutaneous lymphoma, LDH is also used as a tumor marker [13]. Ca125 is a non-specific marker, mainly used for ovarian cancer [14]. The laboratory findings led to the diagnosis of GCT, particularly for the tumor in the sacrococcygeal region.

The diagnosis should be confirmed by FNAB to assess the cytopathology, followed by a microscopic exam of the cell block. The findings in this case support the diagnosis of YST. YST has multiple morphological patterns, according to the literature. Patterns include microcystic, endodermal sinus, solid, myxomatous, papillary, hepatoid, macrocytic, poly-vesicular vitelline, glandular (primitive endodermal), and alveolar-glandular. The most common pattern is the reticular microcystic pattern, which is formed by the vacuolated cytoplasm of tumor cells and looks like a honeycomb under a microscope [14]. Schiller-body Duvall's, capillaries lined by columnar tumor cells, is another pathognomonic finding in YST. The Schiller-Duvall body was not present in our sample. The absence of follicular pattern or diagnostic Reed-Sternberg cells, also ruled out lymphomas. Sacrococcygeal mature teratoma can also be ruled out because cytopathology showed no mixture of mature cells from different germ layers. Polymorphic cells with vacuolated cytoplasm, large nuclei, and some bizarre, irregular membrane with prominent nucleoli were also found, indicating a malignant nature of the lesion [15]. Due to the absence of the Schiller-Duvall body, confirmation of the diagnosis using an IHC examination is needed. The recommended IHC panel includes anti-AFP antibody as a general germ cell marker. It appears as strongly stained granules, distributed diffusely in YST [8]. The presence of tumor markers such as serum AFP and serum beta HCG, as well as IHC findings, aid in the final diagnosis of a YST [15].

Approximately 80% of YST patients respond well to chemotherapy. In advanced cases, cisplatin-based chemotherapy follows surgical resection. In case of the tumors that had spread, the oncologist recommended chemotherapy. Unresectable cases with metastasis have varying prognoses. Higher AFP levels above 1000 ng/mL are also associated with a worse prognosis [9]. Although a patient responded well to chemotherapy, with rapid normalization of AFP serum levels, a follow-up histopathology exam of the lesion seems reasonable. Unfortunately, the patient in this particular case passed away on the seventh cycle of chemotherapy. The patient most likely passed away due to lung and hepatic metastases.

## Conclusions

In conclusion, we report an infrequent case of sacrococcygeal YST in a two-year-old girl with multiple metastases. When dealing with a sacrococcygeal tumor, YST should always be considered in the differential diagnosis. In pediatric patients with pulmonary metastasis, a GCT should always be suspected. The levels of AFP should be monitored for a period of time. YST can manifest in a variety of clinical scenarios in young patients and should never be overlooked. Our case represents a very rare malignant YST with pulmonary and hepatic metastasis, all within a short period of onset to hospital admission. Laboratory and pathology examinations are especially important to confirm the diagnosis, determine the prognosis, and help to adjust the therapy for YST in children.
